# Development of a neural network model for early detection of creatinine change in critically Ill children

**DOI:** 10.3389/fped.2025.1549836

**Published:** 2025-04-04

**Authors:** Celeste G. Dixon, Eduardo A. Trujillo Rivera, Anita K. Patel, Murray M. Pollack

**Affiliations:** Department of Pediatrics, Division of Critical Care Medicine, Children's National Hospital, George Washington University School of Medicine and Health Sciences, Washington, DC, United States

**Keywords:** acute kidney injury, creatinine, pediatric intensive care unit, machine learning, neural network model

## Abstract

**Introduction:**

Renal dysfunction is common in critically ill children and increases morbidity and mortality risk. Diagnosis and management of renal dysfunction relies on creatinine, a delayed marker of renal injury. We aimed to develop and validate a machine learning model using routinely collected clinical data to predict 24-hour creatinine change in critically ill children before change is observed clinically.

**Methods:**

Retrospective cohort study of 39,932 pediatric intensive care unit encounters in a national multicenter database from 2007 to 2022. A neural network was trained to predict <50% or ≥50% creatinine change in the next 24 h. Admission demographics, routinely measured vital signs, laboratory tests, and medication use variables were used as predictors for the model. Data set was randomly split at the encounter level into model development (80%) and test (20%) sets. Performance and clinical relevance was assessed in the test set by accuracy of prediction classification and confusion matrix metrics.

**Results:**

The cohort had a male predominance (53.8%), median age of 8.0 years (IQR 1.9−14.6), 21.0% incidence of acute kidney injury, and 2.3% mortality. The overall accuracy of the model for predicting change of <50% or ≥50% was 68.1% (95% CI 67.6%−68.7%). The accuracy of classification improved substantially with higher creatinine values from 29.9% (CI 28.9%−31.0%) in pairs with an admission creatinine <0.3 mg/dl to 90.0–96.3% in pairs with an admission creatinine of ≥0.6 mg/dl. The model had a negative predictive value of 97.2% and a positive predictive value of 7.1%. The number needed to evaluate to detect one true change ≥50% was 14.

**Discussion:**

24-hour creatinine change consistent with acute kidney injury can be predicted using routine clinical data in a machine learning model, indicating risk of significant renal dysfunction before it is measured clinically. Positive predictive performance is limited by clinical reliance on creatinine.

## Introduction

Approximately one quarter of children admitted to a pediatric intensive care unit (PICU) will develop acute kidney injury (AKI) during the course of their illness (1). Renal dysfunction can cause fluid overload, electrolyte derangements, altered drug metabolism, and uremia. It is associated with higher risk of mortality and morbidity, prolonged PICU and hospitalization stays, and longer duration of mechanical ventilation (1–4). Children diagnosed with AKI are at higher risk for chronic kidney disease (CKD), hypertension, and cardiovascular disease (5–7). Current diagnosis and management of AKI relies on creatinine as the primary indicator of renal function, yet creatinine is a delayed marker of renal injury (8). It can take 24 h or more from an initial insult for creatinine to significantly rise depending on the extent of renal injury, metabolic rate, degree of fluid overload, and underlying pathology such as shock, infection, or toxin exposure (9–12). Measurement is also limited by the accuracy of creatinine laboratory tests, which depending on the assay used may have as much as 20% error at normal adult creatinine values and up to 50% error at lower values (13–16). This is particularly noteworthy in infants who may have a baseline creatinine of 0.3–0.4 mg/dl; Kidney Disease Improving Global Outcomes (KDIGO) Stage 1 AKI (a 50% increase in serum creatinine) may be within the laboratory margin of error for this population (8, 17). Several novel biomarkers of renal injury have been described, but their use has so far been limited by low specificity for AKI, variable performance in different etiologies of AKI, and limited adoption in clinical practice (18).

Accurate prediction of renal dysfunction could benefit at-risk patients. Earlier recognition of risk of renal dysfunction prior to creatinine change could prompt treatment strategies to reduce the risk of ongoing renal injury and to prevent adverse clinical consequences by limiting nephrotoxic medication exposure, avoiding electrolyte derangements, and adjusting fluid management. It could also reduce time to initiation of renal replacement therapy (RRT), which has been associated with improved outcomes (19). However, the same factors that make renal dysfunction challenging to diagnose in real time also contribute to the difficulty predicting its development and clinical course. Previous pediatric efforts to predict renal dysfunction using traditional statistical methods have been limited by relatively small sample size, reliance on expert consensus for selection of variables, prediction only at time of admission, and variable performance of the prediction models (20–22). There is growing interest in using big data with machine learning techniques to predict risk of renal dysfunction in critically ill children (23–25). We aimed to develop and validate a clinically relevant machine learning model using routinely collected clinical data from a large, multi-center database to predict 24-hour creatinine change in critically ill children during their acute illness. Primary outcome was creatinine change of ≥50%, consistent with KDIGO Stage 1 AKI (8).

## Methods

This study was approved by the Children's National Hospital Institutional Review Board with requirement for informed consent waived. Transparent reporting of a multivariable prediction model for individual prognosis or diagnosis (TRIPOD) guidelines were followed (26).

### Database

Data were collected from Real-World Data™ (RWD) (Oracle Corporation, Austin, TX), a national, de-identified database of US hospital admissions. Hospitals do not have to use Cerner EHR to contribute data to RWD. Available data includes demographic information, admission information (location, hospital unit), clinical variables (vital signs, respiratory support, and diagnostic codes), laboratory values, medications administration records, and hospital outcome. All data are time- and date-stamped.

**Figure 1:**
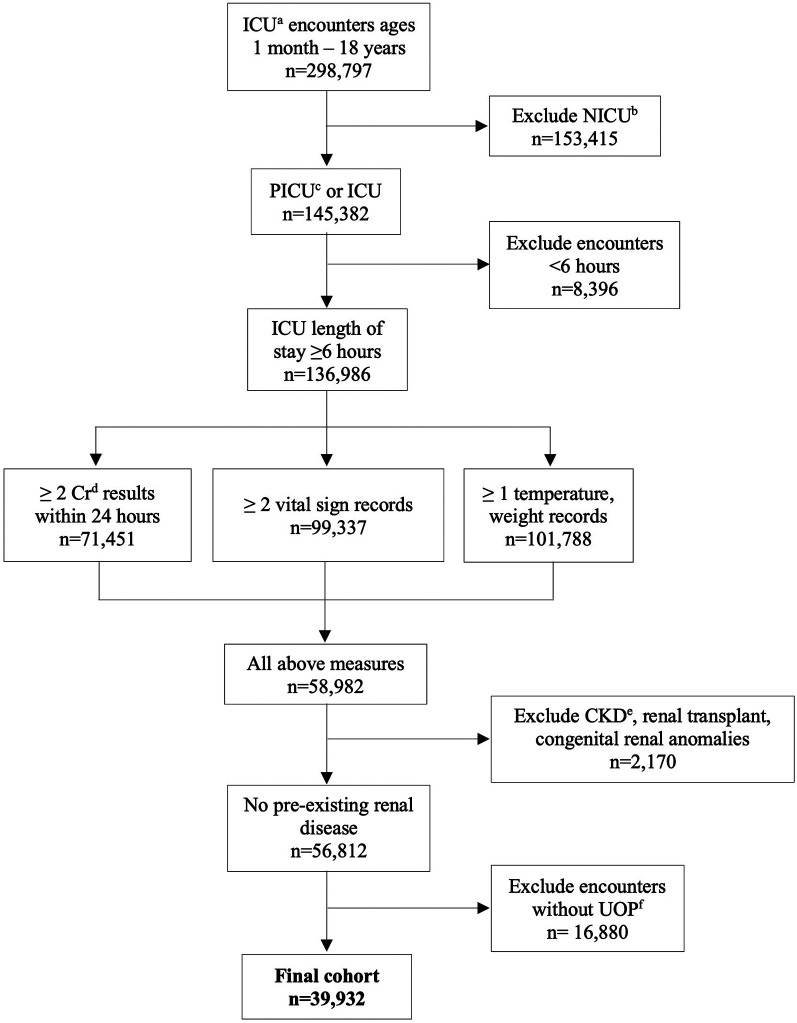
CONSORT diagram of encounter selection. ^a^intensive care unit; ^b^neonatal intensive care unit; ^c^pediatric intensive care unit; ^d^creatinine; ^e^chronic kidney disease; ^f^urine output.

All pediatric encounters (age >1 month to ≤18 years) with an inpatient intensive care unit (ICU) designation were extracted from 2007 to 2022. If repeated ICU admissions occurred within one hospital admission, up to 4 ICU admissions were included (Appendix Table 1). Primary inclusion criteria were a creatinine measured within 24 h of ICU admission (admission creatinine), and at least 2 creatinine values measured within 24 ± 6 h of each other during the ICU admission. Encounters were excluded if ICU length of stay was <6 h, if there were associated diagnostic codes for CKD, congenital renal anomalies, dialysis dependence, or renal transplant, less than 2 records of vital signs, or if there were absent urine output (UOP), weight, or temperature records (Figure 1). Diagnostic codes for CKD, renal anomalies, dialysis dependence, and renal transplant are shown in Appendix Table 6. Other database details are given in Appendices A–E.

### Variables

Demographic and descriptive variables included age, sex, date and time of admission, length of stay, diagnostic codes, and hospital discharge outcome. Clinical event variables included vital signs, respiratory support (fraction inhaled oxygen, positive end-expiratory pressure, peak inspiratory pressure, tidal volume), and urine output. Urine output was expressed as ml/kg/hr computed over 12-hour periods. Laboratory variables included time, date, and result of 43 routinely measured chemistries, blood gases, and hematologic variables (Appendix Table 2). Drug levels were included for gentamicin and vancomycin. Medications included fluids, vasoactives, diuretics, and nephrotoxic medications and were included based on known associations with renal function (27–29). Medications were included as binary variables (received, not received). Missing data was imputed with physiologic normal data until a measurement was recorded (Appendix Tables 3, 4). Variables recorded in less than 500 encounters were excluded. International Classification of Diseases (ICD) −9 and −10 codes were categorized into the following: cardiovascular disease, infection, malignancy, neurologic disease, respiratory disease, trauma/ingestion, other, and not specified, based on respective ICD classifications (30, 31). AKI was defined by KDIGO criteria (8). Detailed descriptions of all variables are given in Appendices B–E.

### Outcome

The primary outcome was a binary prediction of <50% or ≥50% increase in creatinine in the subsequent 24 ± 6 h. The classification threshold of ≥50% was selected to focus on creatinine change meeting AKI criteria (8). This outcome was evaluated for all creatinine pairs occurring in days 1–5 of ICU admission in order to capture changes in renal function in the acute illness phase.

### Model development and performance

Inclusion criteria for data points were that creatinine values had to occur in pairs, with the second value measured 24 ± 6 h after the first. The admission creatinine could serve as the first creatinine of a pair. The second creatinine of a pair could also serve as the first creatinine value for a subsequent pair. All model variables that occurred in the 48 h preceding the first creatinine of a pair were included. These 48 h were divided into 4 12-hour time periods. If there was no recorded value for a variable, the last known result was imputed. The maximum, minimum, average, range, and number of measurements for each variable in each 12-hour period were included. The maximum and minimum value of each variable from the entire admission period preceding the first creatinine of the pair were also included, regardless of when they occurred. If two variables were highly correlated (Pearson correlation value ≥0.9 or ≤−0.9), the most reliable, earliest occurring, directly measured variable was kept and the other removed (see Appendix F).

The data set (141,658 creatinine pairs) was divided randomly into 64% training set, 16% validation set and 20% test set. Random selection was done at the patient level. The training set was used for model development while the validation set was used to avoid overfitting or underfitting during model training. The test sample was used to evaluate model performance. The final model was the result of sequential experimentation using fully connected neural networks. The data were weighted such that positive outcome pairs (≥ 50% increase in creatinine) were weighted 23.6 × the negative outcome pairs. Model parameters used for experimentation were number of hidden layers, number of nodes per hidden layer, smoothing parameter values for L1 and L2 regularizations on the nodes values at each layer, proportion of node dropout per hidden layer, and variable sample weight during training. The validation set mean absolute error was the target for minimization at each training epoch. The mean absolute error and median absolute errors curves for both the training and validation set were used to monitor for overfitting and underfitting. Each model could be trained for 500 epochs, but training was stopped if no reduction on the mean absolute error was observed. The final model had eight hidden layers, with nodes between 114 and 484 per layer. Dropout rates for each layer ranged from 0.01 to 0.19, and L1/L2 smoothing parameters ranged from 0.0001 to 0.002.

Model performance was assessed with accuracy for both the total test sample and compared across groups by admission creatinine, first creatinine of the pair, and age. Performance was further evaluated with confusion matrix metrics and the clinical relevance was assessed with the number needed to evaluate (NNE ± 1/Positive predictive value), the number of cases that need to be screened to identify one at risk case. Accuracy was also compared between predictions made in hours 0–47 and hours 48–120 of PICU admission. The absolute creatinine change was also assessed for all test set creatinine pairs.

Feature importance was evaluated using a Local Interpretable Model-Agnostic Explanation (LIME) approach, which treats each prediction locally as a linear model and assigns covariate importance (32). A random sample of 1000 creatinine pairs where change was ≥50% and 1000 creatinine pairs where change was <50% were used for this analysis. For each case, the 15 most important variables were determined using LIME. The frequency with which each variable appeared in the top 15 variables for the sample cases determined the overall importance.

All model development and analysis was done using R, version 4.3.1 (https://www.r-project.org).

## Results

There were 39,932 encounters in the cohort from 59 unique hospitals (Figure 1), with a male predominance (53.8%), and median age of 8.0 years (IQR 1.9–14.6). Descriptive data are shown in Table 1. Median length of stay was 7.9 days (IQR 4.1–16.3). Overall mortality was 2.3%. There was an associated diagnosis for 88.0% of encounters. The most common categories of diagnoses were respiratory disease (38.0%), cardiovascular disease (24.0%), and infection (23.9%) (Table 1). Incidence of AKI of any stage was 21.0% (8,393 encounters) (Table 1). There were 141,658 paired creatinine measures meeting criteria for model training and testing, of which 5,753 (4.1%) had ≥50% change in creatinine during the 24-hour period. These 5,753 pairs represented 3,637 unique encounters (Table 1). These encounters had a lower median age (4.6 years, IQR 1.0–12.1), higher mortality (6.57%), and longer length of stay (14.2 days, IQR 7.2–28.0).

**Table 1:** Cohort description.

Variable	Total (*n* = 39,932)	≥50% creatinine change (*n* = 3,637)
Male (*n*) (%)	21,500 (53.8%)	1,922 (52.9%)
Median age (years) (IQR)	8.0 (1.9, 14.6)	4.6 (1.0, 12.1)
Mortality (*n*) (%)	813 (2.3%)	239 (6.57%)
Median length of stay (days) (IQR)	7.9 (4.1, 16.3)	14.2 (7.2, 28.0)
AKI any stage (*n*) (%)	8,393 (21.0%)	3,637 (100%)
Associated diagnosis^a^ (*n*) (%)	35,143 (88.0%)	3,181 (87.5%)
Cardiovascular disease	9,572 (24.0%)	1,059 (29.1%)
Infection	9,539 (23.9%)	1,095 (30.1%)
Malignancy	6,077 (15.2%)	634 (17.4%)
Neurologic disease	9,119 (22.8%)	785 (21.6%)
Respiratory disease	15,176 (38.0%)	1,554 (42.7%)
Trauma/ingestion	8,283 (20.7%)	771 (21.2%)
Other^b^	30,221 (86.0%)	2,623 (72.1%)
No diagnosis associated	4,789 (12.0%)	456 (12.5%)

^a^ Diagnostic category associated with encounter.

^b^ Associated diagnosis not included in other diagnostic categories; encounters could have multiple associated diagnoses.

The accuracy of the neural network model is shown in Table 2. Since inclusion of diagnostic code did not improve performance, the final model did not include diagnostic information. The overall accuracy of the model for predicting change of <50% or ≥50% was 68.1% (95% CI 67.6%−68.7%). In the subset of creatinine pairs where the measured change was <50%, the accuracy was 68.7% (CI 68.1% - 69.2%) and for pairs where the measured change was ≥50%, the accuracy was 55.1% (CI 52.2%−57.9%). The accuracy of classification improved substantially with higher creatinine values, from 29.9% (CI 28.9%–31.0%) accuracy in pairs with an admission creatinine <0.3 mg/dl to an accuracy of 90.0%–96.3% in pairs with an admission creatinine of 0.6–≥1.2 mg/dl. Categorized by the first creatinine value of the pair, the accuracy increased from 56.0% (CI 54.9%−57.2%) for creatinine values <0.3 mg/dl to 86.0%–88.9% for those with creatinine 0.6–≥1.2 mg/dl. The accuracy in age groups increased from 51.4% (CI 49.1%–53.7%) in those 1–2 months of age to 85.9% (CI 84.2%–85.6%) for those ≥12 years. Accuracy was higher for predictions made after the first 48 h (73.2%, CI 72.3%−74.0%) than for predictions made in hours 0–47 (65.1%, CI 64.4%−65.8%) of PICU admission.

**Table 2:** Model performance in the test set.

Group	Paired creatinine values count (%)	Accuracy of model (95% CI^a^)
All	27,967 (100)	68.1% (67.6–68.7)
By creatinine change		
<50% Cr^b^ change	26,711 (95.5)	68.7% (68.1–69.2)
≥50% Cr change	1,256 (4.5)	55.1% (52.2–57.9)
By admission creatinine^c^		
<0.3 mg/dl	7,071 (25.3)	29.9% (28.9–31.0)
0.3–0.59 mg/dl	10,603 (37.9)	69.3% (68.4–70.2)
0.6–0.89 mg/dl	5,480 (19.6)	90.0% (89.1–90.8)
0.9–1.19 mg/dl	2,233 (8.0)	94.9% (94.0–95.6)
≥1.2 mg/dl	2,580 (9.2)	96.3% (95.5–96.9)
By first creatinine of prediction pair		
<0.3 mg/dl	6,886 (24.6)	56.0% (54.9–57.2)
0.3–0.59 mg/dl	11,442 (40.9)	71.4% (70.6–72.2)
0.6–0.89 mg/dl	5,767 (20.6)	86.0% (85.1–86.9)
0.9–1.19 mg/dl	1,859 (6.6)	88.1% (86.6–89.6)
≥1.2 mg/dl	2,013 (7.2)	88.9% (87.5–90.2)
By age		
1–2 months	1,753 (6.3)	51.4% (49.1–53.7)
3–5 months	1,563 (5.6)	53.7% (51.2–56.1)
6–11 months	1,689 (6.0)	56.8% (54.4–59.2)
12–17 months	1,249 (4.5)	55.9% (53.1–58.6)
18 months–4 years	4,586 (16.4)	65.3% (63.9–66.7)
5 years–7 years	2,661 (9.5)	76.3% (74.6–77.9)
8 years–11 years	3,641 (13.0)	76.8% (75.4–78.1)
≥12 years	10,825 (38.7)	85.9% (84.2–85.6)

^a^ Confidence interval.

^b^ Creatinine.

^c^ First creatinine measurement, within ± 24 h of admission.

Table 3 shows expanded performance metrics based on admission creatinine. Specificity overall was 68.7% (CI 68.1%–69.2%), with increasing specificity at higher admission creatinine values (92.1%–98.3% when creatinine ≥0.6 mg/dl). Negative predictive value (NPV) was high for all creatinine values (96.5%–98.1%), while positive predictive value (PPV) was low (5.4%–9.3%). The NNE was 14 overall, and ranged from 11 to 19 depending on admission creatinine.

**Table 3:** Expanded model performance.

Group	Sensitivity (95% CI^a^)	Specificity (95% CI^a^)	Negative predictive value (95% CI^a^)	Positive predictive value (95% CI^a^)	Number needed to evaluate^b^
All	55.1% (52.2–57.9)	68.7% (68.1–69.2)	97.2% (97.0–97.5)	7.1% (6.6–7.6)	14
By admission creatinine^c^					
<0.3 mg/dl	85.5% (81.9–88.5)	26.2% (25.2–27.3)	96.5% (95.5–97.2)	7.1% (6.4–7.8)	14
0.3–0.59 mg/dl	49.5% (45.0–54.0)	70.2% (69.3–71.1)	96.8% (96.4–97.2)	7.1% (6.3–8.0)	14
0.6–0.89 mg/dl	15.4% (10.3–22.5)	92.1% (91.3–92.9)	97.4% (96.9–97.8)	5.4% (3.6–8.1)	19
0.9–1.19 mg/dl	16.1% (9.0–27.2)	96.6% (95.9–97.2)	98.1% (97.6–98.6)	9.3% (5.2–16.4)	11
≥1.2 mg/dl	5.4% (1.8–14.6)	98.3% (97.7–98.7)	97.9% (97.3–98.4)	6.5% (2.2–17.5)	15

^a^ Confidence interval.

^b^ Number needed to evaluate = 1/positive predictive value and indicates the number of patients that need to be evaluated to detect one true positive.

^c^ First creatinine measurement, within ± 24 h of admission.

**Figure 2:**
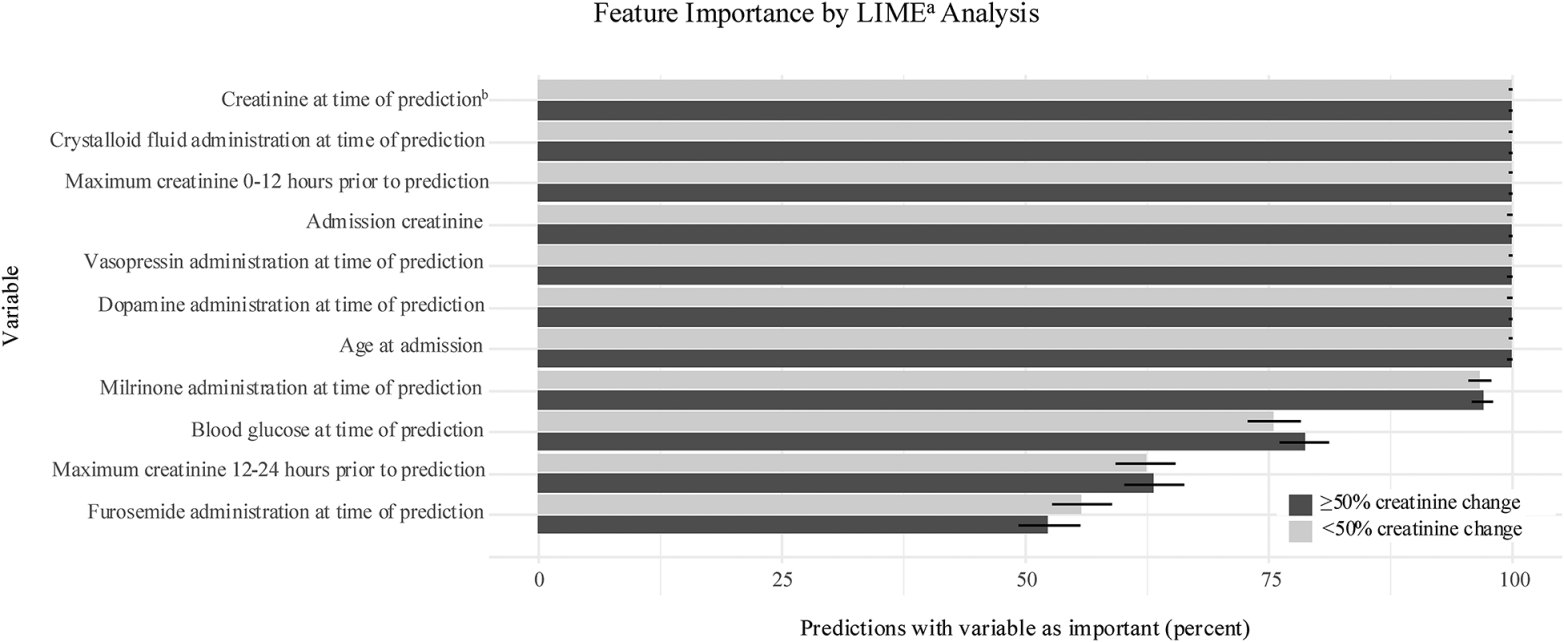
Feature importance. Variables with highest frequency in 15 most important variables for sample predictions. Importance expressed as percent with 95% confidence interval represented by error bar. ^a^Local Interpretable model-agnostic explanation; ^b^first creatinine of prediction pair.

There were seven predictor variables that were among the most important for over 99% of all creatinine pairs (Figure 2). These were present creatinine value (first creatinine value of the pair), maximum creatinine value 0–12 h before prediction, admission creatinine value, crystalloid fluid administration, dopamine administration, vasopressin administration, and age. Several other variables were important for over 50% of predictions, including blood glucose level, creatinine 12–24 h before prediction, furosemide administration, and milrinone administration.

The absolute creatinine change by admission creatinine and the initial creatinine in the creatinine pairs is shown in Table 4. For all groups, the median change for those < 50% creatinine change was no change or a creatinine decrease while there was a substantial absolute change for the creatinine values in all ≥50% creatinine change groups, ranging from 0.16 mg/dl in the lowest admission creatinine group to 0.90 mg/dl in the highest group.

**Table 4:** Creatinine change in outcome groups by admission creatinine or first creatinine in the test set.

Group	Count (*n* = 27,967)	Median actual creatinine change (IQR^a^) (mg/dl)
Admission creatinine^b^ and outcome group		
<0.3 mg/dl		
<50% Cr^c^ change	6,618	0.00 (−0.04, 0.02)
≥50% Cr change	453	0.16 (0.10, 0.20)
0.3–0.59 mg/dl		
<50% Cr change	10,109	−0.01 (−0.10, 0.03)
≥50% Cr change	494	0.25 (0.19, 0.40)
0.6–0.89 mg/dl		
<50% Cr change	5,293	−0.03 (−0.11, 0.02)
≥50% Cr change	187	0.32 (0.19, 0.59)
0.9–1.19 mg/dl		
<50% Cr change	2,182	−0.10 (−0.20, 0.00)
≥50% Cr change	51	0.43 (0.27, 1.12)
≥1.2 mg/dl		
<50% Cr change	2,509	−0.17 (−0.48, 0.00)
≥50% Cr change	71	0.90 (0.21, 1.60)
First creatinine of prediction pair^d^ and outcome group		
<0.3 mg/dl		
<50% Cr change	7,941	0.00 (−0.02, 0.03)
≥50% Cr change	763	0.17 (0.11, 0.20)
0.3–0.59 mg/dl		
<50% Cr change	10,576	−0.01 (−0.10, 0.02)
≥50% Cr change	291	0.30 (0.26, 0.41)
0.6–0.89 mg/dl		
<50% Cr change	4,872	−0.09 (−0.18, 0.00)
≥50% Cr change	96	0.59 (0.47, 0.92)
0.9–1.19 mg/dl		
<50% Cr change	1,562	−0.20 (−0.35, −0.05)
≥50% Cr change	36	0.88 (0.66, 1.23)
≥1.2 mg/dl		
<50% Cr change	1,759	−0.37 (−0.77, 0.00)
≥50% Cr change	69	1.33 (1.00, 1.74)

^a^ Interquartile range.

^b^ First creatinine measurement, within ± 24 h of admission.

^c^ Creatinine.

^d^ First creatinine of creatinine pair

## Discussion

Our results demonstrate that 24-hour creatinine change in critically ill children can be predicted with routine clinical data using a machine learning model. In patients with admission creatinine values of 0.6 mg/dl or higher, this model accurately classified a ≥50% change in creatinine in 90% of cases. The clinical suspicion of renal dysfunction in this sample was, presumably, relatively high since all patients had at least two creatinine values measured. Yet even in this population, only 4.1% of creatinine pairs in the data set had a creatinine change ≥50% in a 24-hour period. This likely contributed to the high NPV and low PPV. Low prevalence of the primary outcome may result in models that can accurately predict negative cases but have low PPV. However, the NNE indicates this model could still be clinically useful. The NNE averaging 14 patients and the high NPV could improve clinical care by identifying most of the low risk patients and isolating a relatively high risk population with a manageable NNE to be screened to detect one true positive. Given the potential clinical impact of renal dysfunction, this model would still be effective in identifying a high risk, but uncommon outcome.

Attempts to predict AKI in critically ill children have been limited by small sample size, restricted variable inclusion, and single time point prediction. The Renal Angina Index (RAI) relies on 5 variables to predict AKI 72 h after admission (20). In validation, only 30% of patients who developed AKI had a positive RAI score (21). The Pediatric Early AKI Risk Score uses 7 variables to predict the same outcome, with good negative predictive performance but lower positive prediction (22). Both studies had less than 10,000 patients. In contrast to these traditional approaches a recent machine learning model was developed in 16,863 patients, which predicted 41% of AKI episodes occurring in the first 48 h of admission with a true positive to false positive of 1:1 (29). Our model had larger sample size, more complete variable inclusion, and better predictive performance with predictions at any point in the first five days of ICU admission. The use of a neural network and outcome of creatinine change also distinguish our model from most other pediatric machine learning applications (33).

Renal dysfunction is challenging to predict due to the complex physiology of renal failure and the limitations of current mechanisms for its evaluation. Renal function is a dynamic process that is impacted by perfusion, oxygenation, systemic inflammation, fluid balance, and nephrotoxin exposure. These parameters may change rapidly in critically ill patients, leading to significant changes in renal function over time. Creatinine is also a delayed marker of renal injury, is confounded by fluid overload, and can have a variable baseline in patients with low muscle mass or chronic illness (9, 34, 35). However, clinical practice still relies on serum creatinine, for monitoring renal function. Several biomarkers of renal injury show promise as better indicators of renal function, but have limited availability and lack consensus on their use in the diagnosis or staging of AKI (36). None were measured with sufficient frequency to be included in our model. Ideally, model performance could be improved in the future by incorporating biomarkers of renal injury. A biomarker that can be measured with more precision and reflect more immediate changes in renal function than creatinine would result in an improved model.

Creatinine assays are particularly unreliable in pediatric patients, which adds to the challenge of an accurate prediction model in infants and young children. Assays may have up to 50% error in samples with creatinine <0.4 mg/dl, and there remains significant inter-assay variability (15, 16). In children with a baseline creatinine of 0.4 mg/dl, a 50% change in creatinine is an absolute change of only 0.2 mg/dl, which makes the detection of true AKI vs. artifact difficult for infants and young children (37). This may account for lower performance metrics, especially the low specificity in the subset of our cohort with low creatinine values. Given the inaccuracy of measured creatinine <0.4 mg/dl, a model that relies on creatinine measurement may be inaccurate at these values. It is possible that the observed difference in model performance is due to the limits of creatinine assays. Indeed, renal dysfunction models developed in adult populations have better performance than pediatric models, which may be a result of higher baseline creatinine values with more reliable laboratory measurements (38–40).

Our model was specifically designed as a dynamic prediction model with multiple variables to account for the complex, rapidly changing physiology during acute illness. This model has the potential to be clinically useful if integrated into the EHR so that predictor variables could be directly used to alert providers to predicted 24-hour creatinine change. The model performs well to predict patients with <50% creatinine change, which may be clinically useful. Identifying patients with low risk of developing renal dysfunction may impact decision making for nephrotoxic therapies such as empiric vancomycin or escalation of diuretics. Additionally, the model can identify cases with risk of ≥50% creatinine change with a NNE of 14. Predicting a rise in creatinine in this context, before it is measured clinically, could give clinicians a window of opportunity in which to mitigate renal damage, adjust fluid and electrolyte management, and reconsider the use of nephrotoxic medications. While working within the limitations of creatinine as a delayed indicator of renal function, it could still be possible to make proactive changes in management to protect the kidneys. The feature importance analysis revealed several medication exposures that were significant in the prediction model, which may indicate opportunities changes in management. It is also notable that performance of our model was not improved with the addition of diagnosis, implying that renal dysfunction can be predicted based on routine clinical measures, independent of the mechanisms of disease. EHR model integration would be an important future direction for this study to evaluate real-time clinical utility.

Our model has several limitations. First, it was developed and tested in a retrospective cohort which spanned a 15-year time period, in which there likely was variation in clinical practice and reliability of measured creatinine. Presumably, there was significant variability in the laboratory measurement techniques by institution and over time, especially influencing the model in the lower creatinine ranges. This extended time period may also explain why dopamine exposure had strong feature importance, as it was used frequently in the past but its use has declined more recently. Second, there are limitations common to the use of large, retrospective database studies. We were restricted to previously collected data, which may bias the study toward sicker patients who are more likely to have creatinine measured. Third, as required for many machine learning models, imputation was required for missing data. Fourth, there are additional limitations inherent to a neural network, including limited interpretability of variable importance. Lastly this model was optimized for predictive performance but has not been implemented clinically. As this model was created on a multicenter database, recalibration at individual sites may optimize its performance (41). The low prevalence of the positive outcome in this model also contributes to its lower performance predicting positive cases. The incorporation of alternate markers of renal dysfunction should be investigated in the future.

In summary, our study shows that short-term creatinine change can be predicted in critically ill children using routinely collected clinical data before a measured rise in creatinine is observed. If the creatinine value either at admission or any time in days 1–5 of PICU admission is ≥0.6 mg/dl, creatinine change in the next 24 h can be accurately predicted. The low prevalence of positive cases in this cohort limits model performance. If this model were applied clinically, 14 patients would need to be screened to detect one positive case. Despite the complexity of creatinine dynamics, the ability to predict creatinine change may allow for clinical interventions that minimize ongoing renal damage, avoid worsening of renal function, and mitigate clinical consequences.

## Data Availability

The original contributions presented in the study are included in the article/Supplementary Material, further inquiries can be directed to the corresponding author/s.

## Appendix

### Appendix A: Overview, data cleaning, and data definitions

Data cleaning: Cleaning of extracted data included removing duplicate or null values, standardizing units of measurement, and removing invalid entries (e.g., negative values, data extremes inconsistent with life). Data cleaning specific to each data type is outlined in sections below.

#### Data definitions

Pediatric patients: >1 month to ≤18 years at time of admission.

Inpatient: Inpatients were defined as those with Real World Data (RWD) encounter classification of “Inpatient”.

Intensive care unit (ICU): All encounters were included within the “Inpatient” classification with a RWD location classification of “intensive care unit” (ICU) or “pediatric intensive care unit” (PICU). “Neonatal intensive care unit” (NICU) encounters were excluded. Encounters with <6 h ICU length of stay were excluded. Up to 4 repeat ICU admission for any given encounter were included, with each ICU admission treated as a distinct event.

**Appendix Table 1:** Repeat ICU admissions.

	Number of encounters (%)
First ICU admission	39,932 (100)
Second ICU admission	491 (1.2)
Third ICU admission	101 (0.3)
Fourth ICU admission	36 (0.1)

### Appendix B: Laboratory variables

Tests included were collected from whole blood or serum, at any site (venous, arterial, or capillary). For each included laboratory test, all recorded units and distribution of results were examined. Units were standardized to the most commonly used unit (ex: mg/dl to g/dl).

The distribution of each laboratory test was analyzed individually, with both descriptive statistics and graphical display. Lower and upper limits were based on clinically relevant ranges and physiology (see below). Values outside of these limits were excluded. Tests with units that did not fit the clinical distribution were also excluded. Any laboratory test that was present in less than 500 encounters was excluded and is not shown. The table below shows the laboratory variables included, the measurement units evaluated and standardized units, and upper and lower limits for range testing.

**Appendix Table 2:** Standardized laboratory variables.

Variable	Units included	Units excluded	Standard units	Lower limit	Upper limit
Alanine transaminase	IU/L, enzyme U/L, IU/ml, g/dl, mg/dl, null	none	U/L	0	2,000
Albumin	g/dl, mg/dl, µg/dl, null	ratio, ml	g/dl	0	10
Ammonia	µmol/L, µg/dl, mmol/L, null	none	µmol/L	0.1	8
Amylase	Enzyme U/L, IU/L, null	none	U/L	0	1,000
Absolute neutrophil count	10^3^/µl, 10^3^, 10^9^/L, µl, kiloenzyme unit/L, mm^3^, cells/µl, kg/m^3^, null	percent	10^3^/µl	0	60
Anion gap	mmol/L, mEq/L, g/dl, mg/dl, null	percent, ratio	mEq/L	0	30
Aspartate aminotransferase	IU/L, enzyme U/L, IU/ml, null	ratio	U/L	0	2,000
Bands	10^3^/µl, 10^3^, 10^9^/L, cells/µl	percent, null	10^3^/µl	0	60
Base excess	mmol/L, meq/L, null	mg/dl, mmHg, percent	mmol/L	−30	30
Bicarbonate (HCO3/total CO2)	mmol/L, µmol/L, mEq/L, mmol, null	percent, °C, mm, mmHg	mmol/L	1	80
Bilirubin direct	mg/dl, g/dl, null	none	mg/dl	0	30
Bilirubin indirect	mg/dl	None	mg/dl	0	40
Bilirubin total	mg/dl, g/dl, null	none	mg/dl	0	40
Blood urea nitrogen	mg/dl, null	meter	mg/dl	0	150
C-reactive protein	mg/dl, mg/L, null	none	mg/dl	0	50
Calcium	mg/dl, mg/ml, null	mmol/L, mg/day	mg/dl	1	15
Calcium ionized	mmol/L, µmol/L, mg/dl, mEq/L, null	mg, mmHg, percent	mmol/L	0	2
Chloride	mmol/L, µmol/L, mEq/L, null	mmol	mmol/L	70	140
Creatinine	mg/dl, mg, null	ml/min, g/24 h, ml/min/173*10^−2^ *m^2^	mg/dl	0.1	10
Creatine kinase	Enzyme U/L, IU/L, null	ng/ml	U/L	0	500,000
Erythrocyte sedimentation rate	mm/hr, null	None	mm/hr	0	150
Fibrinogen	Mg/dl, null	None	mg/dl	0	1,200
Gamma-glutamyl transferase	Enzyme U/L, IU/L, null	enzyme U/ml,	U/L	0	2,000
Gentamicin level	µg/dl, µg/ml, null	None	µg/ml	0	20
Glucose	mg/dl, mg/ml, null	ng/ml, ml/dl, mg/day, ng/dl, mmol/dl, mmol/L,	mg/dl	10	800
Hematocrit	Percent, null	mmHg, mmol/L, ng/ml	percent	1	80
Hemoglobin	gm/dl, mg/dl, g/ml, null	Percent, mmol/mol Cr	gm/dl	1	25
International normalized ratio (INR)	ratio, international normalized ratio, null	none	ratio	0	5
Lactate (arterial)	mmol/L, mEq/L, null	mmHg, percent, nmol/ml, µmol/L, mg/dl	Mmol/L	0	30
Lipase	Enzyme U/L, IU/L, enzyme U/dl, mIU/ml, null	none	U/L	0	3,000
Magnesium	mg/dl, mEq/L, mmol/L, mg/L, null	None	mg/dl	0.1	5
Partial thromboplastin time (PTT)	Seconds, null	none	seconds	1	250
PCO2	mmHg, null	Mmol/L, g/dl, percent, mg	mmHg	1	150
pH	pH, enzyme units, null	None	pH	6.0	8.5
Phosphate	mg/dl, null	none	mg/dl	0.1	10
Platelet count	10^3^/µl, 10^3^, 10^9^/L, µl, kiloenzyme unit/L, mm^3^, kg/m^3^, null	fL, g/dl, 10^6^/µl, percent	10^3^/µl	0	1,200
PO2 (arterial)	mmHg, null	Percent, meter, ml/dl	mmHg	5	600
Potassium	mEq/L, mmol/L, mmol/dl, mmol, µmol/L, null	none	mmol/L	0	12
Procalcitonin	ng/ml, mg/dl, µg/ml	none	ng/ml	0	30
Prothrombin time (PT)	Seconds, null	Percent, µg/ml	seconds	1	80
Sodium	mEq/L, mmol/L, mmol, µmol/L, mmol/dl, null	none	mmol/L	115	180
Vancomycin level	µg/dl, µg/ml, mg/dl, null	None	µg/ml	0	60
White Blood Cell Count	10^3^/µl, 10^3^, 10^9^/L, µl, Kiloenzyme Unit/L, Mm^3^, Cells/µl, 10^6^/Ml, Null	Kg/M^3^	10^3^/µl	0	200

#### Imputed laboratory values

Imputed values were used for variables without a recorded measurement. These were maintained until replaced by a recorded value. When possible, values were imputed based on numerical computation. For variables with insufficient data to use this method, imputed values were selected assuming normal physiology and reference values as follows:.

**Appendix Table 3:** Imputed laboratory values.

Variable	Imputed value [sourced (42, 43), unless otherwise specified]
Ammonia	28 µmol/L
Amylase	73 U/L
Bands	0%
Bilirubin direct	0.35 mg/dl
Bilirubin indirect	1.0 mg/dl
Calcium	9.6 mg/dl
C-reactive protein	1.0 mg/ml
Creatine kinase	170 U/L
Erythrocyte sedimentation rate	10 mm/hr
Fibrinogen	330 mg/dl
Gamma-glutamyl transferase	23 U/L
Gentamicin level	0 µg/ml if no gentamicin given 0.5 µg/ml if gentamicin given (44)
Lactate (arterial)	0.5 mmol/L
Lipase	68 U/L
PCO_2_	38 mmHg
pH	7.40
PO_2_ (arterial)	97 mmHg
Procalcitonin	0.1 ng/ml (45)
Vancomycin level	0 µg/ml if no vancomycin given 10 µg/ml if vancomycin given (46)

### Appendix C: Vital signs, clinical variables, and measurements

Clinical variables and measurements were assessed as follows: all vital signs, clinical values, and measurements in this cohort was collected. All recorded units and distribution of results were examined. Units were standardized to the most commonly used. Units were converted to the standard. Data that could not be converted were excluded.

The distribution of each variable was analyzed individually, with both descriptive statistics and graphical display. Lower and upper limits were set based on clinical medicine. Values outside of these limits were excluded. Tests with units that did not fit the clinical distribution were also excluded. Any clinical variable that was recorded in less than 500 encounters was excluded.

Urine output data was standardized as follows: All measurements with recorded units of ml were included. Amount of urine recorded in a 12-hour period was summed (from 7 AM to 7 PM, and 7 PM to 7 AM). 12-hour urine output was divided by patient weight and hours to obtain urine output in ml/kg/hr for that time period.

Tidal volume for mechanically ventilated patients was also divided by patient weight to obtain tidal volume in ml/kg.

#### Imputed clinical values

As with laboratory values, imputed values were entered for variables without a recorded measurement. These were maintained until replaced by a recorded value. For variables with insufficient data to use this method, imputed values were selected assuming normal physiology values as follows:

**Appendix Table 4:** Imputed clinical values.

Variable	Imputed values
Central venous pressure	5 mm Hg (47)
Peak inspiratory pressure	−8 cm H_2_O (48)
PO intake	0 ml
Tidal volume	8 ml/kg body weight (49)

### Appendix D: Medication variables

All records of crystalloid or colloid volume expanders were evaluated, including those with supplemental electrolytes as listed below (Appendix Table 5). Both bolus and maintenance fluid volumes were counted. Vasoactive medications in both bolus doses and continuous infusions were included. Nephrotoxic medications were selected based on clinical evidence (27–29).

**Appendix Table 5:** Medications.

Medications	
Acetazolamide	Dopamine
Acyclovir	Enalapril
Aspirin	Epinephrine
Bumetanide	Furosemide
Captopril	Gentamicin
Chlorothiazide	Ibuprofen
Colloid fluids	Iodinated contrast media
Albumin 5%	Diatrizoate meglumine/diatrizoate sodium
Albumin 25%	Iohexol
Dextran	Ioversol
Hydroxyethyl starch	Ketorolac
Crystalloid fluids	Milrinone
Isolyte	Norepinephrine
Lactated ringers (+ dextrose)	Phenylephrine
Normosol	Piperacillin-tazobactam
Plasma-lyte	Pentamidine
Sodium chloride 0.9% (+ dextrose, KCl, KH_2_PO_4_, NaHCO_3_)	Spironolactone
Sodium chloride 0.45% (+ dextrose, KCl, KH_2_PO_4_, NaHCO_3_)	Topiramate
Sodium chloride 0.33% (+ dextrose, KCl, KH_2_PO_4_, NaHCO_3_)	Vancomycin
Sodium Chloride 0.225% (+ Dextrose, Kcl, KH_2_PO_4_, Nahco_3_)	Vasopressin

### Appendix E: Diagnosis codes

Diagnostic and procedural codes from the International Classification of Diseases (ICD) −9 and −10 (30, 31) were available for 88% of patients. All diagnostic and procedural codes were first screened for indication of chronic kidney disease, congenital renal abnormalities, renal transplant status, and chronic dialysis dependence (Appendix Table 6). Encounters with these associated codes were excluded from the model.

**Appendix Table 6:** Excluded diagnostic codes.

Description	ICD−9 codes	ICD−10 codes
Chronic kidney disease	403, 404, 582, 585	E08.2, E09.2, E10.2, E11.2, E13.2, I12, I13, N03, N11, N18, N26
Congenital renal abnormalities	753	P96.0, Q60, Q61, Q62, Q63
Absent kidney	55.4, 55.5, V45.73	Z90.5
Renal transplant status	55.6, 996.81, V42.0	T86.1, Z48.22, Z94.0
Chronic dialysis dependence	39.95, 54.98, 996.56, 996.68, 996.73, E879.1, V45.1, V56	I95.3, R88.0, T82.4, T85.611, T85.621, T85.631, T85.691, T85.71, Y84.1, Z49, Z91.15, Z99.2

The diagnoses associated with each encounter were categorized into seven groups: cardiovascular disease, infection, malignancy, neurologic disease, respiratory disease, trauma/ingestion, or other. Diagnostic categories were not mutually exclusive (multiple diagnoses could be associated with a single encounter). If a diagnosis was not associated with a given encounter, it was coded as “no diagnosis associated”.

### Appendix F: Model development

#### Model structure

Every encounter was required to have an admission creatinine measured within the 24 h preceding admission through day 1 of ICU admission. Subsequent creatinine values utilized in the modeling had to occur in pairs separated by 24 ± 6 h. The first creatinine value of a pair defined hour 0–1 of that modeling period, and could occur at any point in days 1–5 of ICU admission. A second creatinine had to be measured within 24 ± 6 h of the first in order to be used for model training or validation. The 48 h preceding the first creatinine of the pair were then divided into 4 12-hour time periods. The number of modeling periods for any given encounter was therefore dependent on the number of creatinine pairs that fit the above criteria.

### Appendix Figure 1: Model structure

For each 12-hour period preceding the first creatinine of the pair, the following metrics were applied to all variables prior to inclusion in the model:

(1) For all vital signs (heart rate, respiratory rate, blood pressure, temperature, oxygen saturation, and GCS), the minimum value, maximum value, average, and count (number of records) were included.
(2) For all other clinical measurements (weight, respiratory support variables), the minimum value, maximum value, and count were included.
(3) For intake and output measurements (UOP) which had been standardized to units of ml/kg/hr over 12 h, a single value was included.
(4) For laboratory values, the minimum value, maximum value, and count were included.
(5) Each medication was included as a binary variable (received/did not receive).

For all variables, the maximum value and minimum value at any point in admission prior to the first creatinine of the pair were also included.

#### Treatment of highly correlated variables

For all variable pairs with Pearson correlation value ≥0.9 or ≤−0.9, the following rules were applied to select one variable of the pair for inclusion in the model:

(1) If the same variable was correlated across two time periods (regardless of parameter), the variable from the earliest time period was kept.
(2) If the same variable was correlated within the same time period, the maximum value was kept.
(3) If the count parameter of different variables were correlated in the same time period, the following hierarchy was applied: measurements > events > labs > medications
  a. If both variables were labs, drug levels were kept over other labs
  b. If both variables were labs but one was a directly measured value and the other was a calculated value, the directly measured lab was kept
(4) If the minimum or maximum parameter of different variables were correlated within the same time period, the following hierarchy was applied: labs > measurements > events > medications
  a. If both variables were labs, drug levels were kept over other labs
  b. If both variables were labs but one was a directly measured value and the other was a calculated value, the directly measured lab was kept
